# 2-(4-Bromo­phen­yl)-4-(4-meth­oxy­phen­yl)-6,7,8,9-tetra­hydro-5*H*-cyclo­hepta­[*b*]pyridine

**DOI:** 10.1107/S1600536813013913

**Published:** 2013-05-25

**Authors:** Ísmail Çelik, Mehmet Akkurt, Hayreddin Gezegen, Canan Kazak

**Affiliations:** a Department of Physics, Faculty of Sciences, Cumhuriyet University, 58140 Sivas, Turkey; b Department of Physics, Faculty of Sciences, Erciyes University, 38039 Kayseri, Turkey; c Department of Physics, Faculty of Arts and Sciences, Gaziosmanpaşa University, 60240 Tokat, Turkey; d Department of Physics, Faculty of Arts and Sciences, Ondokuz Mayıs University, 55139 Samsun, Turkey

## Abstract

In the title compound, C_23_H_22_BrNO, the cyclo­heptane ring adopts a chair conformation. The pyridine ring makes dihedral angles of 58.63 (15) and 8.27 (16)° with the benzene rings. The dihedral angle between the benzene rings is 56.68 (17)°. The crystal packing features C—Br⋯π inter­actions [Br⋯centroid distances= 3.813 (2) and 3.839 (2) Å; C—Br⋯centroid = 126.25 (10) and 138.31 (10)°, respectively, forming a three dimensional supramolecular architecture.

## Related literature

For the biological and pharmacological properties of pyridine-based heterocycles, see: Aida *et al.* (2009 ▶); Ceylan & Gezegen (2008 ▶); Cundy *et al.* (1997 ▶); El-borai *et al.* (2012 ▶); Gezegen *et al.* (2010 ▶); Girgis *et al.* (2007 ▶); Hatanaka *et al.* (2005 ▶); Khidre *et al.* (2011 ▶); Laine-Cessac *et al.* (1997 ▶); Menegatti *et al.* (2006 ▶); Musiol *et al.* (2007 ▶); Rajanarendar *et al.* (2012 ▶). For ring conformation analysis, see: Cremer & Pople (1975 ▶).
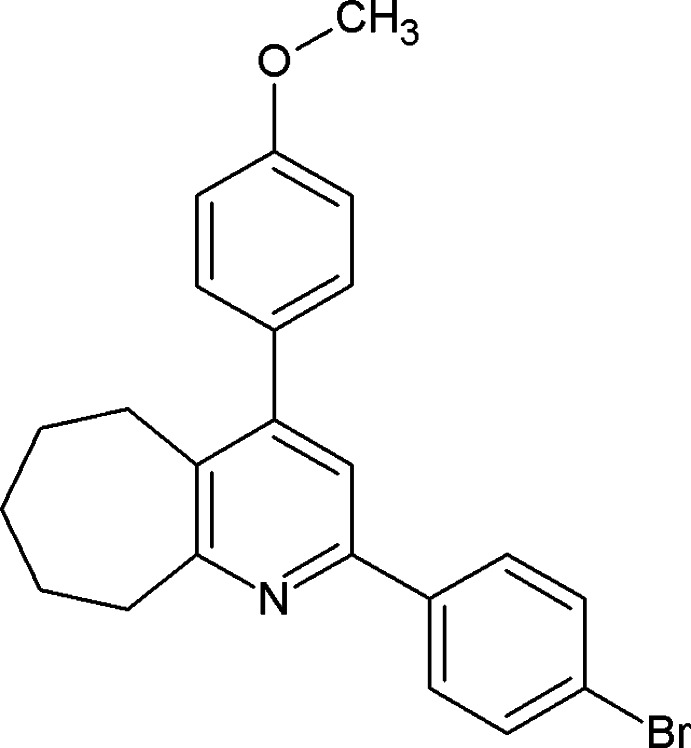


## Experimental

### 

#### Crystal data

C_23_H_22_BrNO
*M* *_r_* = 408.32
Monoclinic, 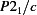

*a* = 10.409 (5) Å
*b* = 10.054 (5) Å
*c* = 18.428 (5) Å
β = 94.850 (5)°
*V* = 1921.6 (14) Å^3^
*Z* = 4
Mo *K*α radiation
μ = 2.15 mm^−1^
*T* = 296 K
0.58 × 0.42 × 0.26 mm

#### Data collection

Stoe IPDS 2 diffractometer
Absorption correction: integration (*X-RED32*; Stoe & Cie, 2002 ▶) *T*_min_ = 0.355, *T*_max_ = 0.572
28207 measured reflections
3986 independent reflections
3083 reflections with *I* > 2σ(*I*)
*R*_int_ = 0.178

#### Refinement

*R*[*F* ^2^ > 2σ(*F* ^2^)] = 0.060
*wR*(*F* ^2^) = 0.119
*S* = 1.12
3986 reflections
235 parameters
H-atom parameters constrained
Δρ_max_ = 0.67 e Å^−3^
Δρ_min_ = −0.42 e Å^−3^

### 

Data collection: *X-AREA* (Stoe & Cie, 2002 ▶); cell refinement: *X-AREA*; data reduction: *X-RED32* (Stoe & Cie, 2002 ▶); program(s) used to solve structure: *SHELXS97* (Sheldrick, 2008 ▶); program(s) used to refine structure: *SHELXL97* (Sheldrick, 2008 ▶); molecular graphics: *ORTEP-3 for Windows* (Farrugia, 2012 ▶); software used to prepare material for publication: *WinGX* (Farrugia, 2012 ▶) and *PLATON* (Spek, 2009 ▶).

## Supplementary Material

Crystal structure: contains datablock(s) global, I. DOI: 10.1107/S1600536813013913/rz5059sup1.cif

Structure factors: contains datablock(s) I. DOI: 10.1107/S1600536813013913/rz5059Isup2.hkl

Supplementary material file. DOI: 10.1107/S1600536813013913/rz5059Isup3.cml

Additional supplementary materials:  crystallographic information; 3D view; checkCIF report

## Figures and Tables

**Fig. 1.**
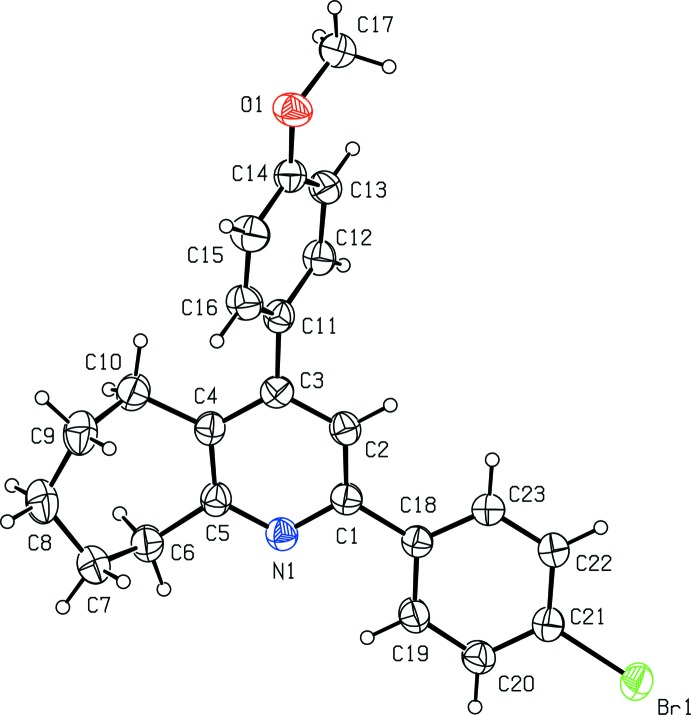
The molecular structure of the title compound with displacement ellipsoids drawn at the 30% probability level.

**Fig. 2.**
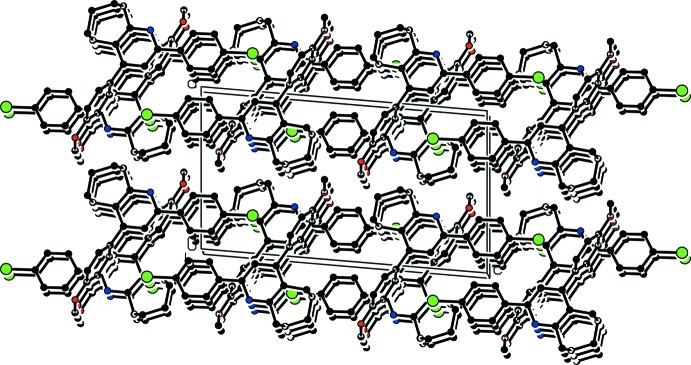
Packing diagram of the title compound viewed down the b axis.

## supplementary crystallographic information

### Comment

Pyridine based heterocycles are found in structure of many natural and biologically important compounds (Aida *et al.*, 2009; Laine-Cessac *et al.*, 1997; Musiol *et al.*, 2007). Also they are known to exhibit numerous biological and pharmacological properties such as antimicrobial (El-borai *et al.*, 2012), antifungal (Khidre *et al.*, 2011), antiviral (Cundy *et al.*, 1997), anticancer (Rajanarendar *et al.*, 2012), antioxidant (Hatanaka *et al.*, 2005), anti-inflammatory (Girgis *et al.*, 2007) and analgesic (Menegatti *et al.*, 2006) activities. In this paper we report synthesis of 2-(4-bromophenyl)-4-(4-methoxyphenyl)-6,7,8,9-tetrahydro-5*H*-cyclohepta[*b*]pyridine with high yield and its crystal structure.

The molecular structure of the title compound (I) is shown in Fig. 1. The cycloheptane ring (C4–C10) of (I) adopts a chair conformation [the puckering parameters (Cremer & Pople, 1975) are Q(2) = 0.422 (4) Å and φ(2) = 311.3 (5)°]. The pyridine ring (N1/C1–C5) makes dihedral angles of 58.63 (15) and 8.27 (16)° with the two benzene rings (C11–C16 and C18–C23), respectively, which have a dihedral angle of 56.68 (17)° between them.

The crystal packing is stabilized by C—Br···π interactions (C21—Br1···*Cg*1^i^: Br···A = 3.813 (2) Å, D—H···A = 126.25 (10)°; C21—Br1···*Cg*2^ii^: Br···A = 3.839 (2) Å, D—H···A = 138.31 (10)°; *Cg*1 and *Cg*2 are the centroids of the N1/C1–C5 and C11–C16 rings, respectively; symmetry codes: (i) x, 1/2-y, 1/2 + z; (ii) -x, -y, 1-z). Fig. 2 represents the molecular packing viewed down the *b* axis.

### Experimental

2-(4-Bromophenyl)-4-(4-methoxyphenyl)-6,7,8,9-tetrahydro-5*H*-cyclohepta[*b*]pyridine was synthesized from the 1,5-diketone derivative (Ceylan & Gezegen, 2008) according to the literature method (Gezegen *et al.*, 2010). To a solution of 2-[3-(4-bromophenyl)-1-(4-methoxyphenyl)-3-oxopropyl]cycloheptanone (1 mmol) in EtOH-AcOH (20 ml 1:1 *v*/*v*) was added NH_4_Ac (4 mmol) and the mixture refluxed for 6 h. After completion of the reaction, the solvent was removed under vacuum and the residue was extracted with CH_2_Cl_2_ (2 × 25 ml). The organic layer was dried over Na_2_SO_4_ and evaporated. The crude product was purified by column chromatography (on a silica gel) eluting with hexane/CHCl_3_ (3:1 *v*/*v*). The obtained brown product was crystallized from EtOH to give pure 2-(4-bromophenyl)-4-(4-methoxyphenyl)-6,7,8,9-tetrahydro-5*H*-cyclohepta[*b*]pyridine in 95% yield; m. p. = 396–398 K.

### Refinement

H-atoms were positioned geometrically and refined using a riding model with C—H = 0.93–0.97 Å, and with *U*_iso_(H) = 1.2 or 1.5*U*_eq_(C). Crystals of the title compound were of limited quality. Even though it was possible to select a specimen suitable for X-ray diffraction experiment, the data obtained were rather poor and the value of R_int_ remained high (17.8%).

### Crystal data

**Table:**

C_23_H_22_BrNO	*F*(000) = 840
*M**_r_* = 408.32	*D*_x_ = 1.411 Mg m^−^^3^
Monoclinic, *P*2_1_/*c*	Mo *K*α radiation, λ = 0.71073 Å
Hall symbol: -P 2ybc	Cell parameters from 3507 reflections
*a* = 10.409 (5) Å	θ = 2.0–29.7°
*b* = 10.054 (5) Å	µ = 2.15 mm^−^^1^
*c* = 18.428 (5) Å	*T* = 296 K
β = 94.850 (5)°	Prism, yellow
*V* = 1921.6 (14) Å^3^	0.58 × 0.42 × 0.26 mm
*Z* = 4	

### Data collection

**Table:**

Stoe IPDS 2 diffractometer	3986 independent reflections
Radiation source: sealed X-ray tube, 12 x 0.4 mm long-fine focus	3083 reflections with *I* > 2σ(*I*)
Plane graphite monochromator	*R*_int_ = 0.178
Detector resolution: 6.67 pixels mm^-1^	θ_max_ = 26.5°, θ_min_ = 2.0°
ω scans	*h* = −13→13
Absorption correction: integration (*X-RED32*; Stoe & Cie, 2002)	*k* = −12→12
*T*_min_ = 0.355, *T*_max_ = 0.572	*l* = −23→23
28207 measured reflections	

### Refinement

**Table:**

Refinement on *F*^2^	Primary atom site location: structure-invariant direct methods
Least-squares matrix: full	Secondary atom site location: difference Fourier map
*R*[*F*^2^ > 2σ(*F*^2^)] = 0.060	Hydrogen site location: inferred from neighbouring sites
*wR*(*F*^2^) = 0.119	H-atom parameters constrained
*S* = 1.12	*w* = 1/[σ^2^(*F*_o_^2^) + (0.0476*P*)^2^ + 0.6943*P*] where *P* = (*F*_o_^2^ + 2*F*_c_^2^)/3
3986 reflections	(Δ/σ)_max_ < 0.001
235 parameters	Δρ_max_ = 0.67 e Å^−^^3^
0 restraints	Δρ_min_ = −0.42 e Å^−^^3^

### Special details

**Table:**

Geometry. Bond distances, angles *etc*. have been calculated using the rounded fractional coordinates. All su's are estimated from the variances of the (full) variance-covariance matrix. The cell e.s.d.'s are taken into account in the estimation of distances, angles and torsion angles
Refinement. Refinement on *F*^2^ for ALL reflections except those flagged by the user for potential systematic errors. Weighted *R*-factors *wR* and all goodnesses of fit *S* are based on *F*^2^, conventional *R*-factors *R* are based on *F*, with *F* set to zero for negative *F*^2^. The observed criterion of *F*^2^ > σ(*F*^2^) is used only for calculating -*R*-factor-obs *etc*. and is not relevant to the choice of reflections for refinement. *R*-factors based on *F*^2^ are statistically about twice as large as those based on *F*, and *R*-factors based on ALL data will be even larger.

### Fractional atomic coordinates and isotropic or equivalent isotropic displacement parameters (Å^2^)

**Table:**

	*x*	*y*	*z*	*U*_iso_*/*U*_eq_	
Br1	0.22100 (4)	0.14765 (5)	0.68332 (2)	0.0767 (1)	
O1	−0.3941 (2)	0.0596 (2)	0.06337 (14)	0.0741 (9)	
N1	0.3068 (2)	0.0105 (3)	0.32719 (14)	0.0564 (9)	
C1	0.1937 (3)	0.0478 (3)	0.35239 (16)	0.0532 (10)	
C2	0.0825 (3)	0.0575 (3)	0.30651 (17)	0.0562 (10)	
C3	0.0829 (3)	0.0282 (3)	0.23272 (17)	0.0544 (10)	
C4	0.2004 (3)	−0.0098 (3)	0.20640 (17)	0.0553 (10)	
C5	0.3082 (3)	−0.0176 (3)	0.25619 (17)	0.0568 (10)	
C6	0.4390 (3)	−0.0579 (4)	0.2335 (2)	0.0700 (12)	
C7	0.4441 (4)	−0.1991 (4)	0.2031 (2)	0.0798 (16)	
C8	0.3898 (4)	−0.2128 (5)	0.1245 (2)	0.0865 (17)	
C9	0.2484 (4)	−0.1809 (4)	0.1093 (2)	0.0839 (16)	
C10	0.2126 (4)	−0.0375 (4)	0.12674 (19)	0.0721 (14)	
C11	−0.0402 (3)	0.0400 (3)	0.18588 (16)	0.0551 (10)	
C12	−0.1082 (3)	0.1580 (3)	0.18180 (18)	0.0595 (11)	
C13	−0.2265 (3)	0.1698 (3)	0.14134 (18)	0.0601 (11)	
C14	−0.2783 (3)	0.0606 (3)	0.10434 (17)	0.0586 (11)	
C15	−0.2119 (4)	−0.0577 (4)	0.1079 (2)	0.0728 (12)	
C16	−0.0948 (4)	−0.0675 (4)	0.1480 (2)	0.0691 (11)	
C17	−0.4694 (4)	0.1773 (4)	0.0621 (2)	0.0760 (14)	
C18	0.1993 (3)	0.0751 (3)	0.43212 (16)	0.0505 (9)	
C19	0.3097 (3)	0.0476 (4)	0.47628 (18)	0.0647 (11)	
C20	0.3172 (3)	0.0689 (4)	0.55055 (19)	0.0641 (11)	
C21	0.2123 (3)	0.1207 (3)	0.58153 (17)	0.0562 (10)	
C22	0.1016 (3)	0.1529 (4)	0.53894 (18)	0.0658 (11)	
C23	0.0957 (3)	0.1291 (3)	0.46491 (18)	0.0624 (11)	
H2	0.00620	0.08400	0.32510	0.0670*	
H6A	0.50170	−0.05060	0.27530	0.0830*	
H6B	0.46390	0.00400	0.19690	0.0830*	
H7A	0.53320	−0.22860	0.20700	0.0960*	
H7B	0.39660	−0.25760	0.23300	0.0960*	
H8A	0.43830	−0.15480	0.09480	0.1040*	
H8B	0.40390	−0.30340	0.10890	0.1040*	
H9A	0.19910	−0.24060	0.13770	0.1000*	
H9B	0.22330	−0.19800	0.05820	0.1000*	
H10A	0.27760	0.02140	0.10980	0.0870*	
H10B	0.13120	−0.01610	0.09960	0.0870*	
H12	−0.07370	0.23180	0.20690	0.0710*	
H13	−0.27030	0.25050	0.13930	0.0720*	
H15	−0.24640	−0.13170	0.08300	0.0870*	
H16	−0.05130	−0.14840	0.14970	0.0830*	
H17A	−0.54790	0.16370	0.03180	0.1140*	
H17B	−0.42180	0.24920	0.04290	0.1140*	
H17C	−0.48930	0.19860	0.11060	0.1140*	
H19	0.38120	0.01360	0.45550	0.0780*	
H20	0.39230	0.04840	0.57930	0.0770*	
H22	0.03170	0.19020	0.55980	0.0790*	
H23	0.02040	0.14980	0.43630	0.0750*	

### Atomic displacement parameters (Å^2^)

**Table:**

	*U*^11^	*U*^22^	*U*^33^	*U*^12^	*U*^13^	*U*^23^
Br1	0.0721 (2)	0.1010 (3)	0.0569 (2)	0.0059 (2)	0.0055 (2)	−0.0121 (2)
O1	0.0727 (15)	0.0659 (15)	0.0804 (16)	0.0092 (12)	−0.0131 (12)	−0.0115 (12)
N1	0.0601 (16)	0.0502 (14)	0.0594 (15)	0.0013 (12)	0.0077 (12)	−0.0005 (12)
C1	0.0608 (18)	0.0430 (16)	0.0564 (16)	0.0004 (14)	0.0080 (14)	0.0016 (13)
C2	0.0578 (17)	0.0502 (18)	0.0613 (17)	0.0005 (14)	0.0096 (14)	−0.0026 (14)
C3	0.0634 (18)	0.0427 (16)	0.0570 (17)	−0.0029 (14)	0.0039 (14)	0.0017 (13)
C4	0.0655 (19)	0.0487 (17)	0.0528 (16)	−0.0003 (14)	0.0115 (14)	0.0005 (13)
C5	0.0653 (18)	0.0504 (18)	0.0557 (17)	0.0000 (15)	0.0119 (14)	−0.0012 (14)
C6	0.065 (2)	0.081 (2)	0.065 (2)	0.0042 (19)	0.0118 (16)	−0.0063 (18)
C7	0.083 (3)	0.082 (3)	0.076 (2)	0.019 (2)	0.017 (2)	−0.005 (2)
C8	0.094 (3)	0.085 (3)	0.083 (3)	0.008 (2)	0.022 (2)	−0.024 (2)
C9	0.089 (3)	0.090 (3)	0.074 (2)	−0.008 (2)	0.015 (2)	−0.023 (2)
C10	0.073 (2)	0.085 (3)	0.0592 (19)	−0.001 (2)	0.0102 (16)	−0.0013 (18)
C11	0.0635 (18)	0.0493 (18)	0.0532 (16)	−0.0004 (15)	0.0089 (14)	−0.0016 (13)
C12	0.0670 (19)	0.0494 (18)	0.0622 (18)	−0.0037 (15)	0.0066 (15)	−0.0081 (14)
C13	0.0662 (19)	0.0509 (19)	0.0631 (18)	0.0062 (15)	0.0049 (15)	−0.0036 (14)
C14	0.0626 (19)	0.0565 (19)	0.0565 (17)	0.0017 (16)	0.0031 (14)	−0.0044 (14)
C15	0.081 (2)	0.052 (2)	0.082 (2)	0.0008 (18)	−0.0129 (19)	−0.0192 (17)
C16	0.081 (2)	0.0498 (19)	0.075 (2)	0.0078 (17)	−0.0021 (18)	−0.0101 (16)
C17	0.075 (2)	0.080 (3)	0.072 (2)	0.013 (2)	0.0000 (18)	−0.0022 (19)
C18	0.0569 (17)	0.0397 (15)	0.0550 (16)	−0.0012 (13)	0.0060 (13)	−0.0014 (13)
C19	0.0603 (19)	0.071 (2)	0.0634 (19)	0.0084 (17)	0.0082 (15)	−0.0124 (16)
C20	0.0579 (18)	0.070 (2)	0.0637 (19)	0.0051 (17)	0.0006 (15)	−0.0079 (17)
C21	0.0610 (18)	0.0501 (18)	0.0580 (17)	−0.0058 (14)	0.0075 (14)	−0.0049 (13)
C22	0.0625 (19)	0.077 (2)	0.0589 (18)	0.0100 (18)	0.0112 (15)	−0.0016 (17)
C23	0.0607 (18)	0.069 (2)	0.0574 (17)	0.0082 (16)	0.0049 (14)	0.0040 (15)

### Geometric parameters (Å, º)

**Table:**

Br1—C21	1.890 (3)	C20—C21	1.377 (5)
O1—C14	1.367 (4)	C21—C22	1.377 (5)
O1—C17	1.419 (5)	C22—C23	1.381 (5)
N1—C1	1.355 (4)	C2—H2	0.9300
N1—C5	1.340 (4)	C6—H6A	0.9700
C1—C2	1.378 (4)	C6—H6B	0.9700
C1—C18	1.491 (4)	C7—H7A	0.9700
C2—C3	1.392 (4)	C7—H7B	0.9700
C3—C4	1.406 (4)	C8—H8A	0.9700
C3—C11	1.488 (4)	C8—H8B	0.9700
C4—C5	1.390 (4)	C9—H9A	0.9700
C4—C10	1.510 (5)	C9—H9B	0.9700
C5—C6	1.513 (5)	C10—H10A	0.9700
C6—C7	1.529 (6)	C10—H10B	0.9700
C7—C8	1.516 (5)	C12—H12	0.9300
C8—C9	1.509 (6)	C13—H13	0.9300
C9—C10	1.530 (6)	C15—H15	0.9300
C11—C12	1.380 (4)	C16—H16	0.9300
C11—C16	1.383 (5)	C17—H17A	0.9600
C12—C13	1.390 (5)	C17—H17B	0.9600
C13—C14	1.378 (4)	C17—H17C	0.9600
C14—C15	1.374 (5)	C19—H19	0.9300
C15—C16	1.375 (6)	C20—H20	0.9300
C18—C19	1.379 (5)	C22—H22	0.9300
C18—C23	1.390 (4)	C23—H23	0.9300
C19—C20	1.381 (5)		
			
C14—O1—C17	117.6 (3)	C7—C6—H6B	109.00
C1—N1—C5	118.3 (2)	H6A—C6—H6B	108.00
N1—C1—C2	121.2 (3)	C6—C7—H7A	109.00
N1—C1—C18	115.2 (3)	C6—C7—H7B	109.00
C2—C1—C18	123.5 (3)	C8—C7—H7A	109.00
C1—C2—C3	120.9 (3)	C8—C7—H7B	109.00
C2—C3—C4	118.0 (3)	H7A—C7—H7B	108.00
C2—C3—C11	118.5 (3)	C7—C8—H8A	108.00
C4—C3—C11	123.6 (3)	C7—C8—H8B	108.00
C3—C4—C5	117.7 (3)	C9—C8—H8A	108.00
C3—C4—C10	122.2 (3)	C9—C8—H8B	108.00
C5—C4—C10	120.1 (3)	H8A—C8—H8B	107.00
N1—C5—C4	123.9 (3)	C8—C9—H9A	109.00
N1—C5—C6	114.3 (3)	C8—C9—H9B	109.00
C4—C5—C6	121.9 (3)	C10—C9—H9A	109.00
C5—C6—C7	114.2 (3)	C10—C9—H9B	109.00
C6—C7—C8	114.4 (3)	H9A—C9—H9B	108.00
C7—C8—C9	115.9 (3)	C4—C10—H10A	109.00
C8—C9—C10	114.4 (4)	C4—C10—H10B	109.00
C4—C10—C9	114.8 (3)	C9—C10—H10A	109.00
C3—C11—C12	120.8 (3)	C9—C10—H10B	109.00
C3—C11—C16	121.9 (3)	H10A—C10—H10B	108.00
C12—C11—C16	117.2 (3)	C11—C12—H12	119.00
C11—C12—C13	122.0 (3)	C13—C12—H12	119.00
C12—C13—C14	119.3 (3)	C12—C13—H13	120.00
O1—C14—C13	124.7 (3)	C14—C13—H13	120.00
O1—C14—C15	115.9 (3)	C14—C15—H15	120.00
C13—C14—C15	119.5 (3)	C16—C15—H15	120.00
C14—C15—C16	120.5 (4)	C11—C16—H16	119.00
C11—C16—C15	121.6 (4)	C15—C16—H16	119.00
C1—C18—C19	120.4 (3)	O1—C17—H17A	109.00
C1—C18—C23	122.2 (3)	O1—C17—H17B	109.00
C19—C18—C23	117.4 (3)	O1—C17—H17C	109.00
C18—C19—C20	121.9 (3)	H17A—C17—H17B	109.00
C19—C20—C21	119.3 (3)	H17A—C17—H17C	110.00
Br1—C21—C20	119.5 (2)	H17B—C17—H17C	110.00
Br1—C21—C22	120.0 (2)	C18—C19—H19	119.00
C20—C21—C22	120.5 (3)	C20—C19—H19	119.00
C21—C22—C23	119.2 (3)	C19—C20—H20	120.00
C18—C23—C22	121.7 (3)	C21—C20—H20	120.00
C1—C2—H2	120.00	C21—C22—H22	120.00
C3—C2—H2	120.00	C23—C22—H22	120.00
C5—C6—H6A	109.00	C18—C23—H23	119.00
C5—C6—H6B	109.00	C22—C23—H23	119.00
C7—C6—H6A	109.00		
			
C17—O1—C14—C13	−3.6 (5)	C3—C4—C5—N1	−0.9 (5)
C17—O1—C14—C15	176.1 (3)	C4—C5—C6—C7	−62.8 (4)
C5—N1—C1—C2	−0.3 (5)	N1—C5—C6—C7	117.9 (3)
C1—N1—C5—C6	179.9 (3)	C5—C6—C7—C8	79.4 (4)
C1—N1—C5—C4	0.5 (5)	C6—C7—C8—C9	−62.9 (5)
C5—N1—C1—C18	179.0 (3)	C7—C8—C9—C10	61.7 (5)
C2—C1—C18—C19	171.9 (3)	C8—C9—C10—C4	−79.3 (4)
N1—C1—C18—C19	−7.4 (4)	C3—C11—C16—C15	176.5 (3)
N1—C1—C18—C23	172.3 (3)	C12—C11—C16—C15	0.0 (5)
N1—C1—C2—C3	0.5 (5)	C3—C11—C12—C13	−176.8 (3)
C2—C1—C18—C23	−8.4 (5)	C16—C11—C12—C13	−0.2 (5)
C18—C1—C2—C3	−178.7 (3)	C11—C12—C13—C14	0.3 (5)
C1—C2—C3—C4	−0.8 (4)	C12—C13—C14—C15	−0.2 (5)
C1—C2—C3—C11	179.8 (3)	C12—C13—C14—O1	179.4 (3)
C11—C3—C4—C10	2.3 (5)	O1—C14—C15—C16	−179.6 (3)
C11—C3—C4—C5	−179.7 (3)	C13—C14—C15—C16	0.0 (5)
C4—C3—C11—C12	−122.9 (3)	C14—C15—C16—C11	0.1 (6)
C2—C3—C11—C12	56.5 (4)	C1—C18—C19—C20	−178.5 (3)
C2—C3—C11—C16	−119.9 (4)	C23—C18—C19—C20	1.8 (5)
C2—C3—C4—C5	1.0 (4)	C1—C18—C23—C22	179.4 (3)
C4—C3—C11—C16	60.7 (4)	C19—C18—C23—C22	−0.9 (5)
C2—C3—C4—C10	−177.1 (3)	C18—C19—C20—C21	−0.9 (6)
C10—C4—C5—N1	177.3 (3)	C19—C20—C21—Br1	179.1 (3)
C5—C4—C10—C9	65.7 (4)	C19—C20—C21—C22	−1.0 (5)
C10—C4—C5—C6	−2.0 (5)	Br1—C21—C22—C23	−178.2 (3)
C3—C4—C10—C9	−116.2 (4)	C20—C21—C22—C23	1.8 (5)
C3—C4—C5—C6	179.8 (3)	C21—C22—C23—C18	−0.9 (5)
